# Altered Senescence-Associated Secretory Phenotype of Human Osteoblasts from Patients with Osteoporosis Enhances Endothelial Cell Migration and Proliferation In Vitro

**DOI:** 10.3390/biology15110858

**Published:** 2026-05-30

**Authors:** Lisa Oezel, Niklas M. Wergen, Robert Zimmermann, Simeon Popov, Beyza Gürsoy, Jan Peter Grassmann, Nele Benölken, Till Kuebart, Bernd Bittersohl, Vera Grotheer

**Affiliations:** 1 Department of Orthopedics and Trauma Surgery, Medical Faculty and University Hospital Düsseldorf, Heinrich Heine University, 40225 Düsseldorf, Germany; 2 Department of Trauma, Hand- and Reconstructive Surgery, Klinikum Osnabrück, 49076 Osnabrück, Germany; 3 Department of Orthopaedics, Klinikum Bielefeld–Mitte, Medical School and University Medical Center OWL, Bielefeld University, 33604 Bielefeld, Germany

**Keywords:** osteoblasts, senescence, angiogenesis, osteoporosis, IL-6, IL-8, angiogenin

## Abstract

Osteoporosis (OP) is a highly prevalent age-associated inflammatory bone disease that remains underdiagnosed and undertreated despite its substantial global burden. OP is characterized by impaired osteoblast (OB) function, alterations in the extracellular matrix and chronic, low-grade inflammation associated with aging (‘inflammaging’). Initial evidence suggests that the accumulation of senescent cells and their senescence-associated secretory phenotype (SASP) may contribute to disease progression. Additionally, growing evidence indicates a close relationship between osteogenesis and angiogenesis in OP. This study aimed to characterize senescence-associated secretory changes in primary human OBs from donors with OP and to assess their functional impact on endothelial cell behavior. Primary human OBs from donors with OP (n = 15; female: 9, male: 6) and without OP (n = 21; female: 14, male: 7) were analyzed for senescence-associated secretory profiles using ELISA, proteome arrays, and Western blot analysis. The effects of OB-conditioned media on endothelial cell behavior were assessed in endothelial cell migration assays. OBs from donors with OP showed a tendency toward increased senescence-associated features in the β-galactosidase assay, alongside an altered secretory phenotype characterized by increased IL-6, reduced IL-8 and angiogenin levels and decreased expression of extracellular matrix-associated proteins, such as osteopontin, osteonectin, progranulin and thrombospondin-1. Conditioned media from OBs from donors with OP significantly enhanced endothelial cell migration and proliferation in vitro. These findings suggest that OBs from donors with OP exhibit a SASP that may alter the angiogenic microenvironment in the bone.

## Simple Summary

This study investigates how primary human osteoblasts (OBs) from osteoporotic donors contribute to changes in the bone microenvironment through senescence-associated secretory processes. We show that osteoporotic OBs display features consistent with cellular senescence and exhibit an altered secretory profile, including changes in inflammatory mediators and extracellular matrix-associated factors. Functional analyses revealed that OB-derived conditioned media significantly enhance endothelial cell migration and proliferation in vitro. These findings suggest that OB-derived signals may influence angiogenesis in osteoporosis and point toward a dysregulated interaction between bone-forming cells and the vascular system.

## 1. Introduction

Osteoporosis (OP) is an inflammatory bone disease with more than 200 million affected patients worldwide [1]. The risk of suffering from an osteoporotic fracture surpasses the combined risk of stroke, myocardial infarction, and breast cancer in the US [2], and the same applies to the economic burden arising from the treatment costs [3]. The consequences for the affected individual on personal life are high, because patients suffer from pain, fragility fractures, disability, immobilization and long-term care [4], social isolation, depression [5], and even mortality [6,7]. But despite the social significance and the burden on the individual, this disease is underdiagnosed, undertreated, and often underestimated [2,8].

Most current therapeutic approaches focus on inhibiting osteoclast activity [8], largely neglecting the molecular mechanisms driving disease onset and progression. On the molecular level, OP is characterized by an imbalance between the receptor activator of NF-κB ligand (RANK-L) and osteoprotegerin (OPG), leading to increased osteoclast activation and bone resorption [9,10]. But, both factors are secreted by osteoblasts (OBs), which also regulate extracellular matrix synthesis [11] and mineralization [12], thereby maintaining bone homeostasis. Furthermore, reduced OB activity [13] and increased apoptosis [14] further compromise bone integrity in OP. These processes lead to reduced bone density, resulting in an increased occurrence of fragility fractures.

In addition to targeting osteoclast function [15], previous research often relies on rodent models or immortalized cell lines [16,17]. However, animal models frequently fail to mimic human bone biology. Numerous clinical trials have demonstrated that compounds effective in mice often lack efficacy in humans, especially for inflammatory diseases [18,19,20]. Furthermore, there is limited understanding of how physiological, age-related bone loss differs from the pathological degradation seen in OP [21]. Although bone density naturally declines from the age of 30, this does not inevitably result in multifactorial OP [21].

An accelerating factor in these processes is the occurrence of chronic inflammation in advanced age. This mechanism, known as “inflammaging,” is caused not only by systemic inflammation but also by the accumulation of senescent cells in bones [22]. Senescent cells have permanently left the cell cycle but remain metabolically active [22]. These cells develop a senescence-associated secretory phenotype (SASP), characterized by altered secretion of cytokines, chemokines, and extracellular matrix proteins, leading to a pro-inflammatory environment [23].

In this context, another aspect that has gained scientific attention is the tight coupling between osteogenesis and angiogenesis [24,25], as adequate vascularization is a prerequisite for bone formation and remodeling. Notably, angiogenesis is likewise compromised under conditions of chronic inflammation [26,27], further linking inflammatory signaling to impaired skeletal regeneration. The vascular network provides bones with oxygen, nutrients, growth factors, and circulating cells [28], and is involved in all skeletal functions such as development, homeostasis and repair [24,29,30].

In OP, impaired angiogenesis appears to contribute to the development and disease progression. A diminished femur blood supply has been observed in patients with postmenopausal OP, and there is a correlation between blood supply to the lower extremities and lower bone mineral density (BMD) [31,32].

Recent studies have also revealed a direct link between vascular subtypes and altered BMD. Kusumbe et al. defined endothelial cells of types H and L in a mouse model, based on morphological, molecular and functional criteria [24]. Type H endothelial cells are associated with the presence of osteoprogenitor cells, mediate neoangiogenesis in bone and are considered a sensitive biomarker of BMD. A loss of type H cells has been shown to result in reduced BMD [24,25,33], now also observed in human OP [34].

The interplay between osteogenesis and angiogenesis coupling is regulated by local mediators, but the exact mechanisms, especially in human pathological processes, have not been sufficiently investigated to date.

This study aimed to characterize senescence-associated changes in the secretory profile of primary human OBs derived from elderly patients with and without OP, focusing on angiogenesis-related mediators. Given the potential impact of the SASP on vascular processes, we further investigated the functional consequences of these alterations by analyzing endothelial cell proliferation and migration (EA.Hy 926 cell line) in response to OB-conditioned media.

## 2. Material and Methods

All chemicals were obtained from Merck KGaA (Darmstadt, Germany), and cell culture materials were purchased from Cellstar (Greiner Bio-One, Solingen, Germany), if not otherwise mentioned. All experiments were performed under sterile conditions.

### 2.1. Patients

The study was approved by the local Ethics Committee of the Heinrich Heine University (Approval No. 5585R, 18 December 2017). All participants provided their written consent, and the study was performed in compliance with the Declaration of Helsinki. Bone density measurements were performed for all study participants. Participants were divided into two groups: one consisting of patients with OP (BMD T-Score ≤ −2.5) and one consisting of individuals with normal bone density (BMD T-Score > −1). A total of 36 patients were included in the study: 15 patients were included in the OP group (female: 9, male: 6), and 21 patients were included in the control group (female: 14, male: 7). Table 1 summarizes the age and gender distribution. All patients underwent hip replacement after fracture or osteoarthritis at the Department of Orthopedics and Trauma Surgery, University Hospital, Düsseldorf, Germany. The inclusion criteria were an age between 18 and 100 years. The exclusion criteria were the lack of consent to participate in the study or chronic infectious diseases. Supplementary Table S1 provides an overview of key clinical characteristics of the patient cohort, including mean vitamin D levels, surgical indication, and other factors relevant to osteoporosis. All data were pseudonymized prior to analysis. Femoral heads were intraoperatively extracted by experienced surgeons and divided with a bone saw. Tissue samples were deposited in phosphate-buffered saline (PBS) with penicillin (100 Units (U)/mL) and streptomycin (100 µg/mL) and then short-term stored in a refrigerator at 4 °C. Samples were processed within 24 h (h).

Due to the use of primary human OB cultures, as well as limited cell availability and experimental constraints, not all assays could be performed with samples from the entire patient cohort. Therefore, the number of biological replicates (*n*) varies between experiments, as indicated in the respective figure legends. Only samples meeting predefined quality criteria (e.g., successful cell isolation, comparable cell passage, healthy cell morphology and sufficient cell numbers) were included in each analysis. Based on our assessment, the subsets of samples used in the individual experiments were representative of the overall cohort, and no systematic bias related to the reduced sample size is assumed.

### 2.2. Isolation and Cultivation of Human Osteoblasts (OBs) from Femoral Heads

The OBs from the two groups were isolated as published previously [35,36,37]. In short, cancellous bone fragments were removed from the center of the femoral head using a sharp spoon. Trabecular bone fragments were then washed with 25 mL standard cultivation medium [Dulbecco’s Modified Eagle’s Medium (DMEM) supplemented with 2 mM α-glutamine (PAA Laboratories, Pasching, Austria), 100 U/mL penicillin, 100 µg/mL streptomycin (PAA), 1 mM sodium pyruvate and 10% fetal bovine serum (FBS) (Biochrom, Berlin, Germany)]. The bone fragments were incubated with collagenase type IV solution (in standard cultivation medium) for 2.5 h on a tumbling roller at 37 °C. The supernatant of the bone fragments with collagenase solution was removed, washed and again centrifuged at 300 *g* for 5 min (min). The supernatant was removed, and OBs were resuspended using 5 mL standard cultivation medium and seeded in a 75 cm^2^ cell culture flask containing 15 mL medium and incubated at 37 °C and 5% CO_2_ in a humidified atmosphere. OBs were monitored, and the medium was changed twice a week. Upon reaching confluence (70–80%), OBs were split at a ratio of 1:3. All experiments were performed in cell culture passage 3.

### 2.3. β-Galactosidase (β-Gal) Assay of Human OBs

The procedure was performed using the β-Galactosidase Staining Kit from Cell Signaling Technology^®^ (9860S) (Danvers, MA, USA). For this purpose, 1 × 10^5^ OBs were seeded in a 6-well plate overnight. After washing the OBs with PBS, they were fixed for 10–15 min according to the manufacturer’s instructions. The subsequent double washing processes were followed by the addition of the staining fixative. The OBs were incubated with this solution overnight at 37 °C in a dry incubator. The following day, the staining of the lysosomal β-gal could be assessed by microscopy.

### 2.4. Osteogenic Differentiation of Human OBs

Human OBs were differentiated as described previously using standard cultivation medium supplemented with 50 µM α-ascorbate-2-phosphate, 10 mM β-glycero-phosphate, and 0.1 µM dexamethasone for 21 days [38,39]. The medium for osteogenic differentiation was changed twice a week [38]. After 21 days of cultivation, ELISA and Western blot analysis were performed. (see the detailed description below).

### 2.5. Enzyme-Linked Immunosorbent Assays (ELISA) for Quantitative Protein Analysis

ELISA from biotechne (Minneapolis, MN, USA) were used according to the manufacturer’s instructions in order to evaluate the following cytokine expressions: Interleukin (IL)-6 (D6050), IL-8 (D8000C), Angiogenin (DAN00), Chitinase 3-like 1 (CH-3-L1) (DC3L10), Plasminogen Activator Inhibitor (PAI)-1 (DY1786); C-X-C motif chemokine (CXCL)-12 (DY350), CCL-2 (DCP00), Vascular endothelial growth factor (VEGF) (DVE00), Osteoprotegerin (OPG) (DY805).

Human OBs were either seeded 1 × 10^5^/6-Well in 3 mL and incubated for 4 days with standard cultivation medium or were osteogenically differentiated for a period of 21 days, whereby the last medium change took place 4 days before collecting and evaluating samples. Supernatants were aliquoted and stored at −80 °C until analysis.

Supernatants for OPG, CXCL-12, Angiogenin, IL-8 ELISAs were diluted (1:3). For VEGF, supernatants were used in a ratio of 1:1, and IL-6 was diluted (1:4), and CH-3-L1 (1:1000). PAI (1:3–1:5), CCL-2 (1:5) and respective samples were analyzed in duplicate. The optical density **λ** = 450 nm was determined with Victor X3 Multilabel-Reader (PerkinElmer, Shelton, CT, USA). All analyses were performed using independent donor samples.

### 2.6. Western Blot Analysis of Human OBs

To compare protein expression in OBs, Western blot analysis was performed. The Pierce BCA Protein Assay Kit was used to determine protein concentration. Respectively, 5 µg protein (if not otherwise mentioned) was mixed with 5 µL Laemmli buffer (4 × Trisglycin-SDS sample buffer, 252 mmol Tris HCl pH = 6.8, 40% Glycerin, 20% mercaptoethanol, 8% SDS, 0.01% bromphenol blue) and centrifuged (3000 *g*, 5 min at 4 °C), and thereafter denatured at 95 °C. The prepared samples were finally separated according to their size with Mini-Protean^®^ TGX stain-free precast gels (Bio Rad, Krefeld, Germany). The separated proteins were transferred to a nitrocellulose membrane (Roche, Mannheim, Germany) with the Trans-Blot Turbo System (Bio Rad). The membranes were blocked with 5% bovine serum albumin (BSA) and afterwards incubated with the following primary antibodies: Osteopontin (MA5-17180; 1:500), Progranulin (MA1-187; 1:500; 15 µg) (both from Thermo Scientific, Waltham, MA, USA) and Osteonectin (sc-25574; 1:2500), Vitamin D receptor (VDR) (sc-13133; 1:500) (both from Santa Cruz Biotechnologies Inc., Dallas, TX, USA), Decorin (ab137508; 1:2000; 20 µg) (from Abcam, Berlin, Germany), Thrombospondin (antibodies online, Aachen, Germany, ABIN3022979; 1:1000; 15 µg). The antibodies were incubated overnight at 4 °C. Anti-goat PO499, anti-mouse P0477, or anti-rabbit P0448 from DAKO (Waldbronn, Germany) conjugated with horseradish peroxidase (HRP) were used as secondary antibodies (1:10,000) with 0.025% anti-Western marker in TBS-T, which was applied for 1 h at RT. Before and after the secondary antibody incubation, the membranes were washed three times with TBS-T. Antibody binding was visualized with ChemiDoc MP Imaging System and analyzed with Image Lab version 6.0.1 build 34, 2017 Standard Edition (Bio Rad, Hercules, CA, USA)), and according to the manufacturer’s protocol, protein concentration was normalized to total protein. All analyses were performed using independent donor samples.

### 2.7. Migration Assay of EA.Hy 926

In order to analyze the impact of OBs on angiogenesis, migration assays were performed. Therefore, conditioned medium from OBs from the two cohorts was applied to endothelial cells (EA.Hy 926 cell line). Ibidi™ inserts (Thistle Scientific Ltd., Glasgow, UK) were used. The ibidi inserts were placed in 12-well culture plates using sterile tweezers. Cell suspensions were prepared at 10^4^ EA.Hy 926/chamber. After 20 h of incubation with a standard cell culture medium or OB-conditioned media, the inserts were carefully removed, and 400 µL medium was applied. Photo-documentation was performed after 4, 8 and 10 h. Cell migration was assessed using ImageJ Freehand Selection Tools by measuring the area covered with the EA.Hy 926 cells. The results were compared with the 0 h time point.

### 2.8. Proteome Profiler Human XL Arrays

Human OBs from the two cohorts were seeded at a density of 1 × 10^5^/6-Well in 3 mL of standard cultivation medium and incubated for 4 days. According to the manufacturer’s instructions (R&D Systems, Minneapolis, MN, USA), 1.5 mL of the conditioned medium was applied to the Proteome Profiler Human XL Oncology Array membranes. Dot blots were visualized using the ChemiDoc MP Imaging System (BioRad Laboratories, Hercules, CA, USA). ImageJ software (version 6.0.1, build 34, 2017, standard edition, from BioRad Laboratories, Hercules, CA, USA) was used to analyze the pixel density of each spot, which represents the amount of bound cytokine. The value of the negative control was subtracted from all other values. Values were further normalized to the values of the reference spots.

### 2.9. Cultivation of EA.Hy 926 Cell Line

EA.Hy 926 cell line was cultivated in DMEM supplemented with 2 mM α-glutamine (PAA), 100 U/mL penicillin, 100 µg/mL streptomycin (PAA), 1 mM sodium pyruvate, 10 mM Hydroxyethyl-piperazineethanesulfonic acid (HEPES), 1% non-essential amino acids (NEAA) and 10% FBS (Biochrom).

### 2.10. Statistical Analysis

The statistical analysis was performed with GraphPad Prism 8.0 (GraphPad Software Inc., San Diego, CA, USA). The Kolmogorov–Smirnov test was used to assess the normality of the data distribution. Potential outliers were detected using the ROUT method with a false discovery rate (Q) of 1%. Homogeneity of variances was assumed, and appropriate statistical tests were applied accordingly. Comparisons between two groups were performed using a two-tailed Student’s *t*-test, while multiple-group comparisons were analyzed using two-way ANOVA. For analyses involving multiple pairwise comparisons, *p*-values were adjusted using the Benjamini–Hochberg false discovery rate (FDR) procedure. Adjusted *p*-values < 0.05 were considered statistically significant. Data are presented as mean ± standard deviation (SD).

## 3. Results

### 3.1. Increased Senescence-Associated Features in Osteoblasts (OBs) from Osteoporotic Donors

Human OBs from elderly patients were divided into two groups: OBs from donors with OP (T-Score < −2.5) and, as a control group, those from donors with a normal BMD (T-Score > −1). The average age of donors with OP was 78.4 years. Donors in the control group were 69.7 years on average.

The reason for hip replacement differed slightly between the two groups. In the OP group, 66.7% underwent surgery due to a fracture and only 33.3% due to coxarthrosis. In the control group, 38% had a fracture, and 61% had coxarthrosis. To highlight further differences between the two groups, clinical data, including vitamin D levels, calcium, phosphate, GFR, ALP, prevalence of diabetes, smoking status, and glucocorticoid use, are described in Supplementary Table S1. No significant differences were observed between the two groups. However, there was a non-significant trend toward higher alkaline phosphatase (ALP) levels, as expected for patients with OP.

**Figure 1:**
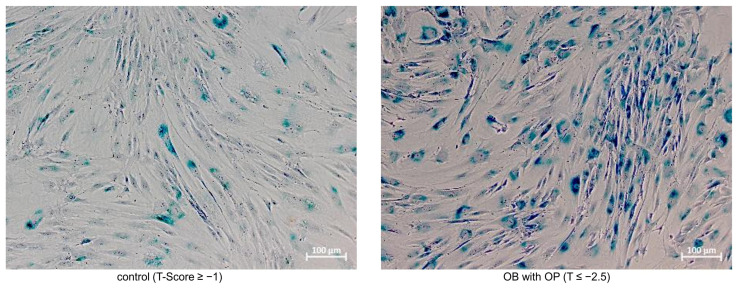
Representative micrographs of OBs derived from the control group and donors with OP following senescence-associated β-galactosidase (β-Gal) staining. OBs from the OP donors exhibited increased β-Gal activity compared to controls, as indicated by more intense blue staining.

To gain an initial understanding of the differences between the two groups, the degree of senescence was determined. For this purpose, a β-galactosidase (β-Gal) assay was performed. OBs from donors with OP showed a tendency toward increased β-Gal activity (Figure 1).

### 3.2. Altered Secretory Phenotype in OBs from Osteoporotic Donors

Following the finding that OBs from patients with OP showed increased senescence-associated features and the knowledge of senescence-associated secretory phenotypes (SASP) with the development of pro-inflammatory conditions, an attempt was made to determine alterations in the expression of potential mediators of bone remodeling, with a focus on factors that affect angiogenesis.

As an initial exploratory approach, supernatants from five donors per group were pooled and analyzed using a cytokine array to screen for differentially secreted proteins (Supplementary Figure S1). Based on this screening, selected factors were further quantified in independent donor samples with ELISA and Western blot.

**Figure 2:**
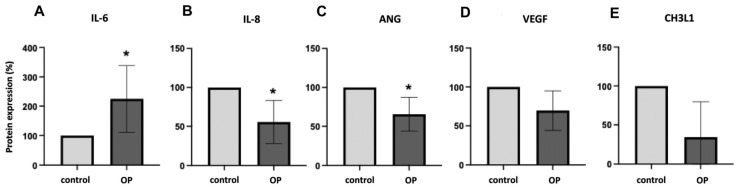
Protein concentrations in conditioned media from OBs derived from the control group and from donors with OP were quantified by enzyme-linked immunosorbent assay (ELISA). Data were normalized to the control group, which was set to 100%. (**A**) IL-6 (control n = 6; OP n = 7; *p* = 0.0458). (**B**) IL-8 (control n = 7; OP n = 8; *p* = 0.0323). (**C**) Angiogenin (ANG) (control n = 8; OP n = 7; *p* = 0.0086). (**D**) Vascular endothelial growth factor (VEGF) (control n = 8; OP n = 7; *p* = 0.9118). (**E**) Chitinase-3-like protein 1 (CHI3L1) (control n = 8; OP n = 8; *p* = 0.2013). IL-6 secretion was increased, whereas IL-8 and ANG were reduced in OBs from OP donors compared with controls (*p* < 0.05). No significant differences were observed for VEGF and CHI3L1. An asterisk (*) indicates statistical significance (*p* < 0.05).

This revealed that IL-6 expression was significantly higher in OBs from patients with OP (Figure 2A; *p* < 0.05), while IL-8 expression was significantly reduced compared to the control group (Figure 2B; *p* < 0.05).

ANG, VEGF, and CH3-L1 were measured (Figure 2C–E). ANG levels were significantly lower in OBs from patients with OP. No statistically significant differences in VEGF and CH-3-L1 expression were observed, although there was a trend toward lower levels of VEGF and CH-3-L1 in OBs from patients with OP.

**Figure 3:**
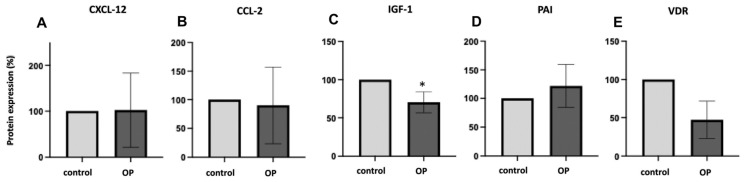
Protein concentrations of (**A**) CXCL-12 (n = 8 per group, *p* = 0.9118), (**B**) CCL2 (control n = 11; OP n = 7; *p* = 0.7505), (**C**) IGF-1 (control n = 8; OP n = 7; *p* = 0.005) and (**D**) Plasminogen activator inhibitor-1 (PAI-1) (control n = 10; OP n = 11; *p* = 0.9118) in conditioned media from OBs derived from the control group and donors with OP quantified by enzyme-linked immunosorbent assay (ELISA). Data were normalized to the control group, which was set to 100%. Vitamin D receptor (VDR) expression was analyzed by Western blot and normalized to total protein (**E**) (n = 7 per group, *p* = 0.0725). No significant differences were observed for CXCL12, CCL2, PAI-1 and VDR between groups. IGF-1 secretion was reduced in OBs from donors with OP compared with controls (*p* < 0.05). An asterisk (*) indicates statistical significance (*p* < 0.05).

CXCL12 and CCL2 were determined (Figure 3A,B). No significant differences in CCL2 and CXCL12 secretion were observed. Furthermore, IGF-1 was monitored, due to its well-known function in promoting endothelial cell migration, tube formation, and VEGF synthesis [40] and as expected, IGF-1 expression was significantly reduced in OBs from patients with OP (Figure 3C).

As it has been shown that PAI-1 is involved in the glucocorticoid-induced decrease in angiogenesis during bone repair [41], PAI-1 was investigated (Figure 3D). No significant differences were observed. A trend toward reduced VDR expression (Figure 3E) was observed in OBs from patients with OP. However, this difference was not statistically significant.

### 3.3. Expression of Extracellular Matrix Proteins in OBs from Osteoporotic Donors

**Figure 4:**
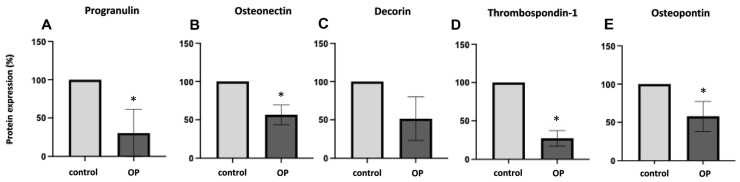
Protein expression of (**A**) Progranulin (control n = 4; OBs n = 8, *p* = 0.0282), (**B**) Osteonectin (n = 5 per group, *p* = 0.0005), (**C**) Decorin (n = 6 per group, *p* = 0.0725), (**D**) Thrombospondin-1 (control n = 4; OBs n = 5, *p* = 0.0005), and (**E**) Osteopontin (n = 6 per group, *p* = 0.0053) in OBs derived from the control group and from donors with OP, analyzed by Western blot. Data were normalized to the control group, which was set to 100%. All analyzed proteins were significantly reduced in OBs from donors with OP compared with controls (*p* < 0.05). An asterisk (*) indicates statistical significance (*p* < 0.05).

Progranulin expression was also analyzed (Figure 4A). Progranulin is a multifunctional growth factor that is known to play a role in tissue regeneration and inflammation [42], and it may also promote angiogenesis [43]. Recent studies have shown that reduced progranulin expression affects bone metabolism by regulating the tumor necrosis factor receptor (TNFR) signaling and the estrogen receptor pathway [42,44]. Consistent with these studies, we detected a significant reduction in progranulin expression in OBs from patients with OP.

We also investigated the altered protein expression of Osteonectin (Figure 4B), Decorin (Figure 4C), Thrombospondin-1 (Figure 4D), and Osteopontin (Figure 4E). Osteonectin, Thrombospondin-1, and Osteopontin expression were significantly reduced in OBs from patients with OP. Decorin expression showed a trend toward lower expression, but this difference was not statistically significant.

### 3.4. Altered Expression Pattern Following Osteogenic Differentiation

OBs from both groups were osteogenically differentiated in cell culture passage 3 for 21 days. The aforementioned factors were then reassessed. While most of the previously observed significant differences were no longer detectable following cultivation and osteogenic differentiation (Supplementary Figures S2 and S3), a significant reduction in CXCL-12 and Osteopontin expression persisted in osteoporotic osteoblasts.

### 3.5. Effects of the Altered Expression Pattern on Endothelial Cell Migration and Proliferation

**Figure 5:**
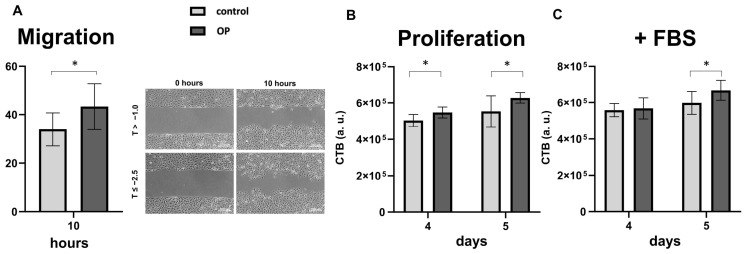
(**A**) Migration of EA.hy926 endothelial cells treated with conditioned medium derived from primary OBs from the control group and from donors with OP assessed by standardized migration assay; representative images of the migration assay and corresponding quantitative analysis are shown (n = 3 per group, after 10 h *p* = 0.0024). (**B**) Proliferation of EA.hy926 cells following prolonged incubation with conditioned medium from OBs from both groups (n = 3 per group, day 4: *p* = 0.0261, day 5: *p* = 0.0006). (**C**) Proliferation of EA.hy926 cells cultured in conditioned medium supplemented with 3% fetal bovine serum (FBS) to exclude nutrient deficiency as a confounding factor (n = 3 per group, day 5: *p* = 0.0009). Conditioned medium from OBs from donors with OP significantly increased endothelial cell migration and proliferation compared with controls (*p* < 0.05). An asterisk (*) indicates statistical significance (*p* < 0.05).

To investigate the impact of the OBs’ secretory phenotype on endothelial cell behavior, EA.Hy 926 cells were treated with conditioned medium derived from primary OBs from both groups. Endothelial cell migration assay was subsequently performed using a standardized migration assay. Surprisingly, there was a significant improvement in the migration potential of endothelial cells treated with OBs from patients with OP (Figure 5A). Furthermore, following prolonged culture of endothelial cells in conditioned medium from OBs from patients with OP, significantly increased proliferation was detected (Figure 5B). Furthermore, the observed effect was not attributable to nutrient deficiency because the medium from the respective OBs (from both groups) was saturated with FBS (3%) (Figure 5C). The results remained the same: Conditioned medium from OBs from patients with OP significantly increased endothelial cell proliferation (Figure 5B).

## 4. Discussion

### 4.1. Senescence-Associated Features in Human Osteoblasts from Elderly Patients with Osteoporosis

Aging is frequently accompanied by life-altering comorbidities, with OP being one of the most prevalent conditions [45]. However, chronological age does not necessarily reflect physiological or biological aging. Long-lived families, for example, often exhibit lower levels of chronic inflammation and a more robust immune response in advanced age [46]. This raises the question whether OBs derived from patients with OP undergo accelerated aging or, more specifically, display an increased level of cellular senescence. Cellular senescence is known to impair proliferation, differentiation, and cellular function, and thereby contribute to accelerated bone loss [47], suggesting that senescent OBs would be involved in the progression of OP [48].

Indeed, multiple cell types within the bone microenvironment have been shown to acquire senescent characteristics with advancing age, including B cells, T cells, myeloid cells, OB progenitors, mature OBs, and osteocytes. Notably, these senescent features were observed partly independently of the senescence-associated secretory phenotype (SASP). The most widely used biomarker for the identification of senescent cells is the senescence-associated β-galactosidase (SA-β-gal) assay, which reflects the increased lysosomal biogenesis that is characteristic of senescent cells [49,50]. Therefore, we compared OBs from patients with OP and OBs from patients with normal bone density. We observed increased SA-β-gal activity in osteoblasts from donors with OP compared to controls, suggesting a tendency toward enhanced cellular senescence (Figure 1). However, it should be noted that SA-β-gal activity is merely one marker of senescence. Cellular senescence is a complex process involving other mechanisms. Further analyses in human OBs from elderly patients with OP, such as telomerase deficiency and shortening, cell cycle arrest mediated via activation of p53/p21^WAF1/CIP1^ and p16^INK4A^/pRB tumor suppressor pathways, and mitochondrial dysfunction [51] would be necessary to further support these observations. It should also be noted that the two groups were not perfectly matched. Given the differences between the two groups, potential age-related effects cannot be ruled out.

However, if this finding is confirmed in further studies, the increased lysosomal activity observed here in OBs with β-Gal assay could be an indication that autophagy, a highly conserved intracellular catabolic process, in which cytoplasmic components are lysosomal degraded for nutrient and/or energy generation, is disturbed and contributes to the pathogenesis of OP. There are a few pointers underlying this: in a rodent model, suppression of autophagy decreased bone loss, associated with aging or estrogen deprivation [52]. In addition, heightening the master autophagy regulator Transcription Factor EB (TFEB) in OBs lineage cells increases bone mass and strength [53]. To get an overview of the function of autophagy in bone homeostasis, Yin’s review provides a comprehensive overview [54].

However, since the objective of our study was to evaluate the secretory phenotype, we did not pursue this interesting observation.

### 4.2. Altered Senescence-Associated Secretory Phenotype (SASP) and the Effect on Angiogenesis

We compared the altered SASP expression pattern in patients with OP to that in our control group. The focus was on factors that also interfere with angiogenic signaling. A summary of the analyzed proteins is presented in Table 2, whereas key factors are discussed in greater detail in the following section:

#### 4.2.1. Interleukin 6 (IL-6)

IL-6 has been described as a component of SASP with a significant impact on angiogenesis [55,56]. The increased secretion of IL-6 in OBs from osteoporotic patients, as shown here (Figure 2), supports previous studies [9,57]. Furthermore, IL-6 mediates OBs differentiation depending on the differentiation stage. In the early stages, IL-6 promotes differentiation [58], while it prevents further differentiation at later stages [59]. In the context of OP, it has been shown that IL-6 leads to increased bone resorption via direct and indirect stimulation of osteoclast formation [60].

IL-6 is a pleiotropic cytokine with complex and context-dependent effects on angiogenesis. Proangiogenic effects have been described for IL-6 via various signaling pathways: IL-6 induces increased expression of VEGF [61] and further enhances angiogenesis by affecting Notch ligands and Angiopoietin-2 [55,62]. In contrast, IL-6 has negative effects on the migratory capacity of endothelial cells. According to Zegeye et al., these negative effects seem to occur primarily in the context of IL-6 trans-signaling [63]. IL-6 trans-signaling induces pro-inflammatory activities, whereas IL-6 cis-signaling provides regeneration and protection [64].

#### 4.2.2. Interleukin 8 (IL-8)

The reduced expression of IL-8 detected in OBs from osteoporotic patients in this study is striking (Figure 2). In the context of “*inflammaging*” and similar to IL-6, it is expected to be upregulated, which would lead to increased osteoclastogenesis and bone resorption. The main function of IL-8 is to attract and activate neutrophils after a (bacterial or viral) infection [65] and to promote tissue repair, blood flow restoration and wound healing [65]. Walsh et al. investigated cytokine expression in human OBs and found no difference in IL-8 mRNA between patients with and without OP [66]. But the examined OBs were isolated from the iliac crest, and it is known that cells from the same tissue origin differ depending on the site of collection [67]; moreover, the evaluated control group was clearly younger [66]. Other studies demonstrated a higher IL-8 level in the blood of OP patients [68,69]. We assume that the elevated chemokine levels may be caused by macrophages, lymphocytes, epithelial cells or endothelial cells, which primarily produce IL-8 [70]. Overall, the effect of IL-8 on bone remodeling and OP is poorly understood [71]. This is mainly due to the lack of an IL-8 counterpart in rodents [72]. This highlights the importance of further studies on human primary cells. Interestingly, an additional IL-8 (100 U/mL) application on endothelial cells inhibited migration, and an inhibition of IL-8 with Reparixin (10^−6^ M) raised migration and diminished OPG expression in OBs.

#### 4.2.3. Angiogenin (ANG)

ANG is a ribonuclease and acts as a proangiogenic factor by promoting the proliferation and migration of endothelial cells. The formation of capillaries and vessels is also supported by ANG [73]. There is little data on the impact of ANG on OP, but a study by Liu et al. suggests that ANG’s importance in bone metabolism may have been underestimated [74]. In their study, the researchers used a mouse model to demonstrate that ANG expression by osteoclasts protects neighboring vascular cells from cellular senescence [74]. They also showed that glucocorticoid therapy inhibits ANG expression in osteoclasts, which ultimately disrupts angiogenesis in the growing skeleton [74].

ANG expression was significantly reduced in osteoblasts (Figure 3), yet conditioned media from osteoporotic osteoblasts enhanced endothelial migration, implying that other soluble factor(s)—not ANG—mediate the altered endothelial cell behavior.

#### 4.2.4. Vascular Endothelial Growth Factor (VEGF)

Interestingly, in our study, a trend toward decreased VEGF expression in OBs from osteoporotic donors was documented (Figure 3D). Apparently, the elevated IL-6 level does not induce VEGF expression in OBs themselves. However, it is important to note that VEGF is highly regulated, and even minor changes in its expression can significantly impact angiogenesis. Our results regarding VEGF contrast with a 2018 study by Veeriah et al., which investigated the effect of OBs on angiogenesis in mimicked disuse OP [75]. The unloaded murine OBs showed increased VEGF expression both in vivo and in vitro and had a positive effect on the migration of endothelial cells (EA.Hy 926). The different results may be due to the different pathogenesis in disuse OP and senile OP, as well as the mouse model used by Veeriah et al. [75].

Interestingly, it could be shown that VEGF dose-dependently promotes bone resorption in osteoclasts [76], possibly the reduced expression is a countereffect to prevent further increased bone resorption, which is characteristic of OP.

Even though we did not find any significant difference in VEGF expression between the OBs from patients with OP and the control group, IL-6 can still have a proangiogenic effect via a VEGF-independent signaling pathway. It has been demonstrated that the proangiogenic effects of IL-6 are linked to a dysfunctional pericytic coverage [55], possibly leading to elevated vascular permeability and instability. Whether this affects the formation of H-vessels in bone is still unclear.

In supplementary investigations of our study, we examined the effect of IL-6, IL-8 and ANG on the migratory capacity and the cell number of EA.Hy 926 cells (Supplementary Figure S4). The results showed a decrease in migration due to IL-6 and IL-8 and an increase in migration with the addition of anti-IL-6 and Reparixin. These results align with those of Zegeye et al.’s study [63]. However, due to IL-6’s pleiotropic and context-dependent effects with pro- and antiangiogenic properties, its impact on angiogenesis in osteoporotic bone remains elusive. As expected, ANG increased the migratory ability of EA.Hy 926 cells, while anti-ANG inhibits migration. In addition, the expression of osteoprotegerin (OPG) in EA.hy 926 cells was assessed following treatment with IL-6, IL-8, ANG and their respective inhibitors. Anti-IL-6, Reparixin and ANG significantly reduced OPG expression in endothelial cells.

**Table 2:** Overview of the selected proteins, including their differential expression in osteoblasts from donors with OP in this study, as well as their reported functional roles in angiogenesis and bone biology.

Protein	Results in This Study (OP vs. Control)	Functional Role in Angiogenesis and Bone Biology
CHI3L1	No significant change	- pro-inflammatory and pro-angiogenic factor associated with “inflammaging” [77,78,79] - has an anabolic effect on bone [80] - mainly expressed by endothelial cells and macrophages [81] - CH-3-L-Knockout-mice show reduced OPG expression and osteoporotic bone loss [80]
CXCL12	No significant change	- is associated with disease severity in postmenopausal OP [82] - effect on the recruitment, development, and survival of human osteoclasts [83] - previous studies report increased expression in osteoclasts and reduced expression in osteoblasts from donors with OP [84] - relevant for angiogenesis in bone, as reduced CXCL12 (together with Osteopontin) has been linked to impaired vascularization and bone healing in experimental models [85]
CCL2	No significant change	- pro-inflammatory cytokine with pro-angiogenic properties [86] - pro-angiogenic properties appear to be mediated by the induction of VEGF [86] - discussed as an early marker of incipient bone loss [87]
IGF-1	↓ decreased	- plays a central role in muscle development and tissue repair [88] - contributes to angiogenesis, at least in part via induction of IL-8 [89] and VEGF [90] - reduced IGF-1 expression may be linked to decreased vitamin D receptor (VDR) levels, as VDR directly regulates IGF-1 transcription [91]
PAI-1	No significant change	- associated with cellular senescence [92] - contributes to glucocorticoid-induced impairment of angiogenesis during bone repair [41] - blocking PAI-1 appears to protect against bone loss in cases of estrogen deficiency [93] - PAI-1 appears to inhibit the VEGF signaling pathway [94]
VDR	No significant change	- Vitamin D is a key regulator of VDR expression [95], and reduced VDR levels are consistent with the commonly observed vitamin D deficiency in osteoporosis - has been shown to be positively coupled with eNOS synthesis in bone [96], suggesting that reduced VDR expression may impair angiogenesis - Vitamin D supplementation in prednisolone-induced OP in rats has resulted in a significant rise in VEGF expression, which is most likely mediated via VDR [97]
Progranulin	↓ decreased	- important regulator of ECM organization and cell–matrix interactions, influencing both bone and endothelial cell function [98,99] - reduced expression of PGRN in OBs from patients with OP was recently described [42,44] - inhibitory effect on tumor necrosis factor α (TNFα) and associated proinflammatory processes [42] - reduced osteoclast differentiation and promoted OBs differentiation [42] - presumably exerts pro-angiogenic effects in endothelial mouse cells [43]
Osteonectin (SPARC)	↓ decreased	- calcium-binding matricellular protein abundantly expressed in mineralized tissues and secreted by osteoblasts during bone formation [100] - modulates angiogenesis via interaction with VEGF and is associated with neovascularization in multiple tissues [101,102,103]
Decorin	No significant change	- regulates ECM assembly and modulates angiogenesis through interactions with IGF-IR, VEGFR2, EGF, ANG, and TGF-β in a context-dependent manner [104,105,106,107,108,109,110,111] - depending on the molecular microenvironment, it can either promote or inhibit angiogenesis [111,112,113]
Thrombospondin-1	↓ decreased	- potent inhibitor of angiogenesis [114], that mediates interactions such as adhesion, migration, and proliferation in endothelial cells [115] - TSP-1 knockout mice displayed increased bone mass, but this was accompanied by reduced biomechanical stability and impaired bone quality [116]
Osteopontin	↓ decreased	- key mediator of cell adhesion and bone matrix mineralization [117,118] - promotes cell survival and proliferation via MAPK signaling pathways [119,120] - exerts pro-angiogenic effects and interacts with VEGF signaling [121,122]

### 4.3. Impact of Osteogenic Differentiation on the Secretory Profiles

Interestingly, most of the previously observed differences in the secretory profile between OBs from OP donors and controls were no longer detectable after 21 days of osteogenic differentiation. However, a significant reduction in CXCL-12 and Osteopontin expression persisted in osteoporotic OBs.

It remains to be determined whether these findings can be confirmed in studies with larger sample sizes. If reproducible, several, not mutually exclusive, mechanisms may explain this observation. First, it is possible that prolonged in vitro culture and differentiation selectively favor subpopulations of OBs with lower senescence-associated features. Second, osteogenic differentiation may activate RUNX2-dependent programs that partially restore matrix production and thereby reduce disease-associated differences.

With regard to pro-inflammatory cytokines, it cannot be excluded that the initially observed differences are influenced by the clinical context (fracture or osteoarthritis), which served as the indication for hip replacement. Both conditions may contribute to a local pro-inflammatory environment. In this context, the convergence of both groups after 21 days of osteogenic differentiation may reflect a partial normalization under standardized in vitro conditions.

### 4.4. Effects of the Supernatant of the OBs on Endothelial Cell Migration and Proliferation

The observation of enhanced endothelial migration and proliferation in response to osteoporotic OBs supernatants (Figure 5) reflects alterations in endothelial cell behavior rather than angiogenesis per se. The assays performed in this study assess specific components of the angiogenic process, such as migration and proliferation, but do not capture vessel formation or functional angiogenesis in vivo.

The increased endothelial responses observed in vitro may indicate a shift toward a pro-migratory and pro-proliferative secretory profile of osteoporotic OBs, potentially as an adaptive response to a hypovascular and senescence-associated microenvironment. In OP, profound alterations of the bone ECM, together with defective vessel stabilization and pericyte coverage, are likely to override pro-angiogenic signaling and prevent the formation of functional vasculature. These regulatory layers are not recapitulated in monocellular culture systems and therefore represent plausible mechanisms underlying the discrepancy between enhanced endothelial motility in vitro and impaired angiogenesis observed in osteoporotic bone in vivo.

However, these effects should be interpreted with caution, as in vitro systems lack the structural and biomechanical complexity of the bone niche. Furthermore, the use of EA.hy 926 cells represents a limitation, as this cell line does not fully reflect the phenotype of primary human endothelial cells or the specialized vascular environment of bone microvasculature.

## 5. Conclusions

Our data indicate that osteoporotic osteoblasts may adopt a secretory phenotype that promotes endothelial cell migration and proliferation in vitro. However, this response appears to be insufficient to restore functional angiogenesis in vivo, where extracellular matrix alterations, impaired vessel stabilization, and disrupted stromal–vascular interactions prevail. Thus, angiogenic failure in osteoporotic bone is unlikely to result from a lack of pro-angiogenic signals alone, but rather from an inability of the altered bone microenvironment to translate these signals into stable, functional vasculature.

It should be noted that the relatively small sample sizes in certain experimental approaches represent a limitation of this study. Therefore, the findings should be interpreted with caution, and mechanistic conclusions remain preliminary. Further studies using larger cohorts and more physiologically relevant models are required to validate these observations.

## Figures and Tables

**Table 1:** Characteristics of the study cohorts, including age and sex distribution (total patients with OP n = 26; total patients with normal BMD n = 18).

	Without OP (BMD T-Score > −1)		with OP (BMD T-Score ≤ −2.5)	
	Female	Male	Female	Male
Total	14	7	9	6
Age (mean)	70	70	82	74
Range	54–83	32–89	61–97	57–94

## Data Availability

The datasets used and/or analyzed during the current study are available from the corresponding author on reasonable request.

## Supplementary Materials

The following supporting information can be downloaded at: https://www.mdpi.com/article/10.3390/biology15110858/s1, Table S1: Clinical parameters of both groups. Values are presented as mean ± SD, and the reason for categorical variables as number (percentage). Group comparisons were performed using a two-tailed Welch’s *t*-test; for categorical variables the Fisher’s exact test was performed. A *p*-value < 0.05 was considered statistically significant. Patients in the OP-Group were older, particularly among females, while no significant differences were observed for most laboratory parameters. There was a non-significant trend toward higher alkaline phosphatase (ALP) levels, as expected for patients with OP. In contrast to the control group, fractures were the most common reason for hip replacement in the osteoporosis group. Figure S1: Proteome Profiler Human XL Arrays. Conditioned media derived from primary OB of donors with OP and a control group were pooled from five donors per group and subjected to semi-quantitative cytokine array analysis to screen for differentially secreted proteins associated with bone remodeling and angiogenesis. Representative array membranes are shown. Differences in spot intensity indicate altered secretion profiles between the two groups. Figure S2: Impact of Osteogenic Differentiation on the Secretory Profiles. OB from donors with OP and a control group were osteogenically differentiated in culture at passage 3 for 21 days. Following differentiation, protein levels (mean ± SD) of the previously analyzed factors were reassessed. (A) IL-6 (T ≥ 1, n = 6; T ≤ 2.5, n = 8; *p* = 0.7099). (B) IL-8 (T ≥ 1, n = 7; T ≤ 2.5, n = 8; *p* = 0.0939). (C) Angiogenin (ANG) (T ≥ 1, n = 8; T ≤ 2.5, n = 6; *p* = 0.4975). (D) Vascular endothelial growth factor (VEGF) (T ≥ 1, n = 8; T ≤ 2.5, n = 6; *p* = 0.413). (E) Chitinase-3-like protein 1 (CHI3L1) (n = 7 per group, *p* = 0.363). No significant differences were detected between the two groups, indicating that osteogenic differentiation normalized the expression of these factors. Figure S3: OB from donors with OP and a control group were osteogenically differentiated in culture at passage 3 for 21 days. Following differentiation, protein levels (mean ± SD) of the previously analyzed factors were reassessed. (A) CCL (T ≥ 1, n = 10; T ≤ 2.5, n = 6; *p* = 0.7736); (B) Progranulin (T ≥ 1, n = 6; T ≤ 2.5, n = 5; *p* = 0.231); (C) Thrombospondin-1 (n = 5 per group, *p* = 0.1354); (D) PAI (n = 5 per group, *p* = 0.1354); (E) CXCL-12 (n = 8 per group, *p* = 0.0005); (F) IGF-1 (n = 8 per group, *p* = 0.2036); (G) Osteopontin (n = 6 per group, *p* = 0.0196); (H) Osteonectin (n = 6 per group, *p* = 0.369). No significant differences were detected for most factors between the two groups following osteogenic differentiation, suggesting a partial normalization of expression; however, selected factors such as CXCL-12 and Osteopontin remained significantly altered. Figure S4: OPG-Expression, cell number and migration of EA.hy926 endothelial cells following stimulation with IL-6, IL-8, ANG and respective inhibitors. (A) Anti-IL-6 (n = 7 per group, *p* = 0.0084), (C) Reparixin (n = 7 per group, *p* = 0.009) and (E) ANG (n = 7 per group, *p* < 0.0001) significantly reduced OPG expression in endothelial cells. (B, D, F) No significant differences in cell numbers were found (n = 7 per group). (G) IL-6 (n = 4 per group, *p* = 0.0192) and (I) IL-8 (n = 4 per group, *p* < 0.0001) treatment reduced cell migration compared with controls. (K) Following stimulation with ANG, migratory capacity increased compared with controls (n = 4 per group, *p* = 0.0378). The respective inhibitors, (H) anti-IL-6 (n = 4 per group, *p* = 0.0884), (J) Reparixin (n = 4 per group, *p* = 0.0805), and (L) anti-ANG (n = 4 per group, *p* = 0.0289), tend to have opposite effects.
