# Supplementary material for: Altered Senescence-Associated Secretory Phenotype of Human Osteoblasts from Patients with Osteoporosis Enhances Endothelial Cell Migration and Proliferation In Vitro
Source: Biology (Basel). 2026 May 30;15(11):858. doi: 10.3390/biology15110858 (PMC13255591; doi:10.3390/biology15110858)
Supplement: Supplementary file 1 [file biology-15-00858-s001.zip › biology-4219265-supplementary.pdf]

Supplementary Table S1

	Control (n = 21)	Osteoporosis (n = 15)	p-value
**Age, years**	69.7 ± 14.1	78.4 ± 12.9	0.06
**Age (female), years**	69.6 ± 9.3	81.7 ± 10	0.01
**Age (male), years**	70 ± 20.1	73.6 ± 16.2	0.72
**eGFR, mL/min/1.73 m²**	68.2 ± 30.1	66.3 ± 28.9	0.85
**Vitamin D, ng/mL**	23.4 ± 17.7	16.5 ± 8.1	0.28
**Calcium, mmol/L**	2.17 ± 0.11	2.21 ± 0.08	0.3
**Phosphate, mmol/L**	1.1 ± 0.38	1.2 ± 0.56	0.63
**ALP, U/L**	80 ± 29.7	141 ± 80.9	0.06
**Diabetes Typ 2, n (%)**	2 (9.5%)	2 (13.3%)	1.0
**Systemic glucocorticoid, n (%)**	1 (4.8%)	2 (13.3%)	0.56
**Smoking status, n (%)**	2 (9.5 %)	1 (6.7%)	1.0
**Reason for hip replacement: Fracture, n (%)**	8 (38%)	10 (66.7%)	0.09
**Reason for hip replacement: Osteoarthritis, n (%)**	13 (61%)	7 (33.3%)	0.09

Clinical parameters of both groups. Values are presented as mean ± SD, and the reason for categorical variables as number (percentage). Group comparisons were performed using a two-tailed Welch's t-test; for categorical variables the Fisher's exact test was performed. A p-value < 0.05 was considered statistically significant. Patients in the OP-Group were older, particularly among females, while no significant differences were observed for most laboratory parameters. There was a non-significant trend toward higher alkaline phosphatase (ALP) levels, as expected for patients with OP. In contrast to the control group, fractures were the most common reason for hip replacement in the osteoporosis group.

Supplementary Figure S1

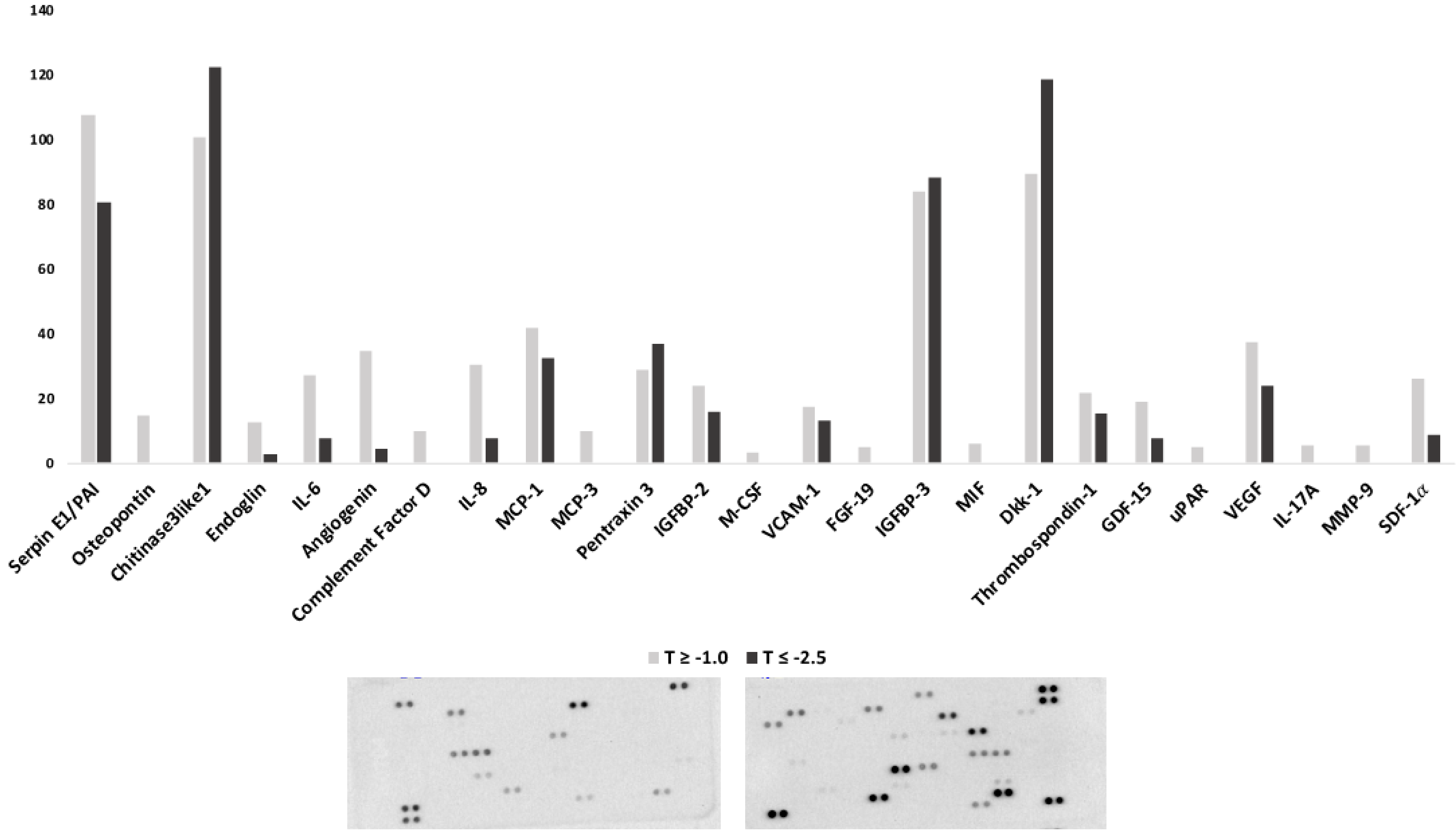

Conditioned media derived from primary OB of donors with OP and a control group were pooled from five donors per group and subjected to semi-quantitative cytokine array analysis to screen for differentially secreted proteins associated with bone remodeling and angiogenesis. Representative array membranes are shown. Differences in spot intensity indicate altered secretion profiles between the two groups.

Supplementary Figure S2

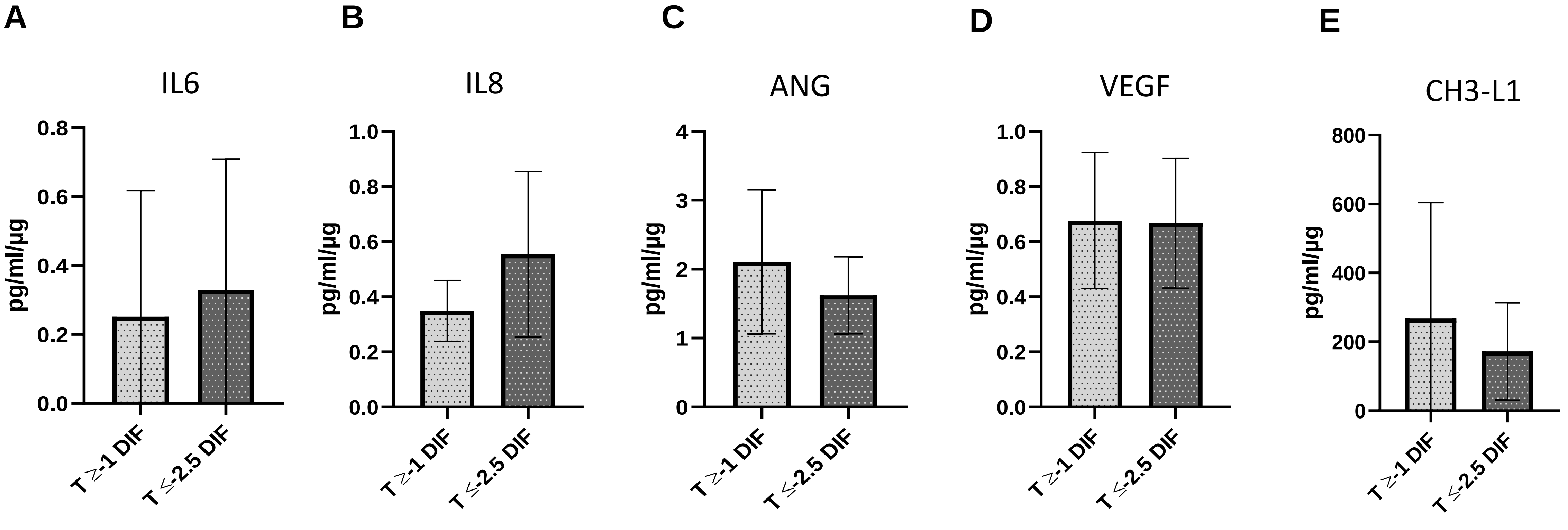

OB from donors with OP and a control group were osteogenically differentiated in culture at passage 3 for 21 days. Following differentiation, protein levels (mean ± SD) of the previously analyzed factors were reassessed. (A) IL-6 (T≥1 n=6; T≤2.5 n=8; p=0.7099). (B) IL-8 (T≥1 n=7; T≤2.5 n=8; p=0.0939). (C) Angiogenin (ANG) (T≥1 n=8; T≤2.5 n=6; p=0.4975). (D) Vascular endothelial growth factor (VEGF) (T≥1 n=8; T≤2.5 n=6; p=0.413). (E) Chitinase-3-like protein 1 (CHI3L1) (n=7 per group, p=0.363). No significant differences were detected between the two groups, indicating that osteogenic differentiation normalized the expression of these factors.

Supplementary Figure S3

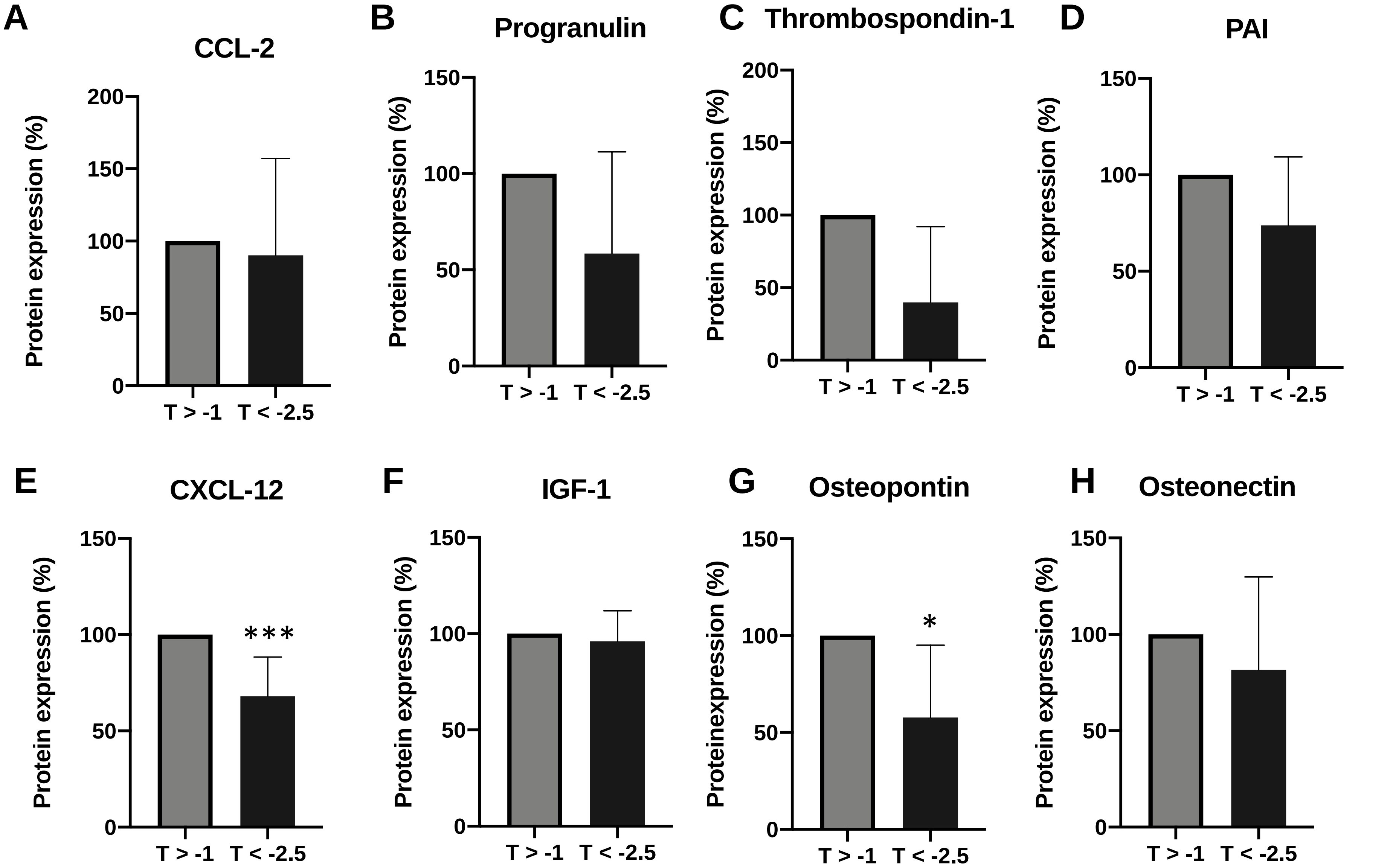

OB from donors with OP and a control group were osteogenically differentiated in culture at passage 3 for 21 days. Following differentiation, protein levels (mean ± SD) of the previously analyzed factors were reassessed. (A) CCL (T≥1 n=10; T≤2,5 n=6; p=0.7736); (B) Progranulin (T≥1 n=6; T≤2,5 n=5; p=0.231); (C) Thrombospondin-1 (n=5 per group, p=0.1354); (D) PAI (n=5 per group, p=0.1354); (E) CXCL-12 (n=8 per group, p=0.0005); (F) IGF-1 (n=8 per group, p=0.2036); (G) Osteopontin (n=6 per group, p=0.0196); (H) Osteonectin (n=6 per group, p=0.369). No significant differences were detected for most factors between the two groups following osteogenic differentiation, suggesting a partial normalization of expression; however, selected factors such as CXCL-12 and Osteopontin remained significantly altered.

Supplementary Figure S4

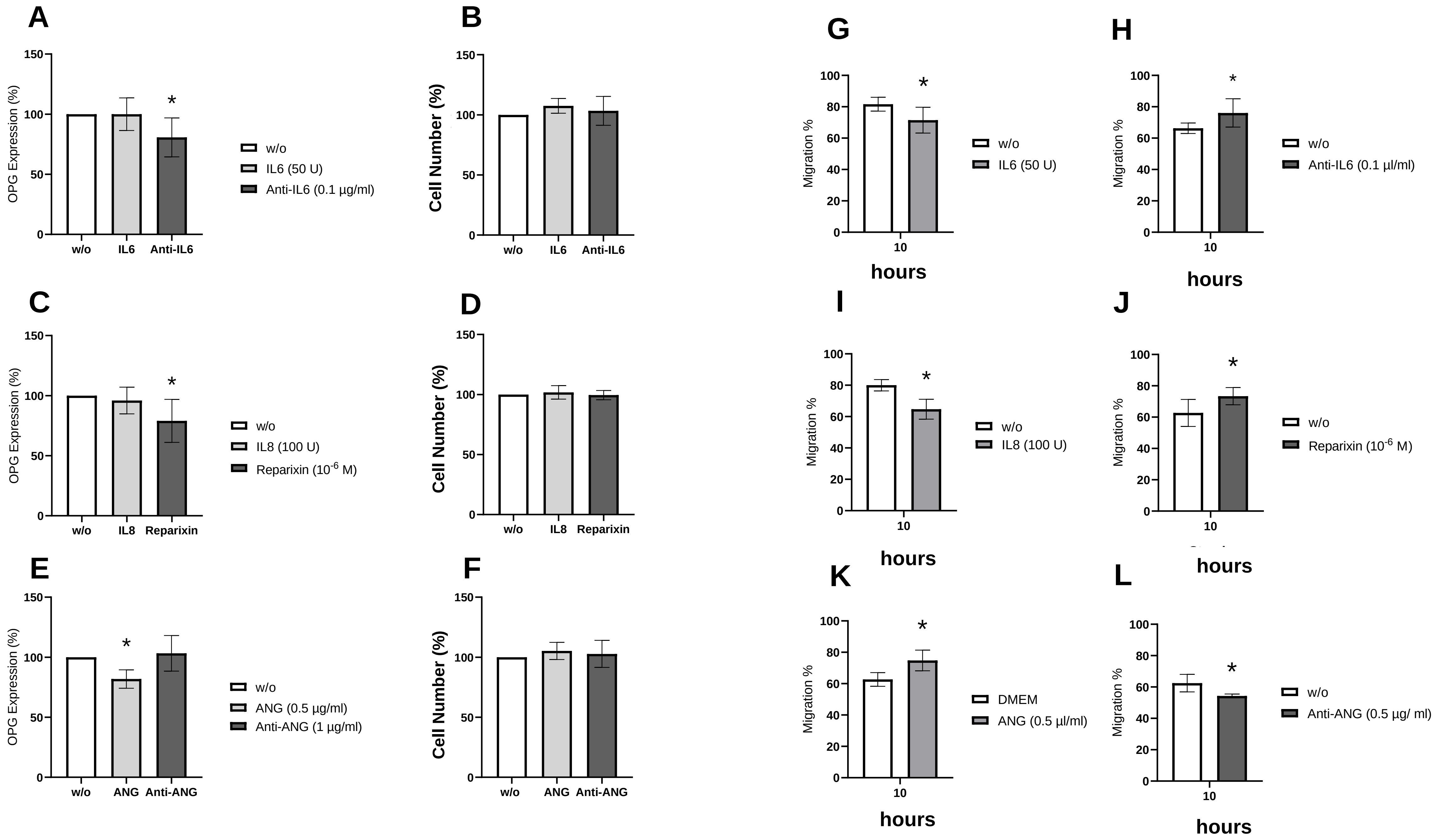

Supplements S4

OPG-Expression, cell number and migration of EA.hy926 endothelial cells following stimulation with IL-6, IL-8, ANG and respective inhibitors. (A) Anti-IL-6 (n=7 per group, p=0.0084), (C) Reparixin (n=7 per group, p = 0.009) and (E) ANG (n=7 per group, p < 0.0001) significantly reduced OPG expression in endothelial cells. (B, D, F) No significant differences in cell numbers were found (n=7 per group). (G) IL-6 (n=4 per group, p=0.0192) and (I) IL-8 (n=4 per group, p < 0.0001) treatment reduced cell migration compared with controls. (K) Following stimulation with ANG, migratory capacity increased compared with controls (n=4 per group, p=0.0378). The respective inhibitors, (H) anti-IL-6 (n=4 per group, p=0.0884), (J) Reparixin (n=4 per group, p=0.0805), and (L) anti-ANG (n=4 per group, p=0.0289), tend to have opposite effects.
